# An Ethnobotanical Study on Qīng-Căo-Chá Tea in Taiwan

**DOI:** 10.3389/fphar.2020.00931

**Published:** 2020-06-25

**Authors:** Shyh-Shyun Huang, Ting-Yang Chen, Jeng-Shyan Deng, Li-Heng Pao, Yung-Chi Cheng, Jung Chao

**Affiliations:** ^1^ School of Pharmacy, China Medical University, Taichung, Taiwan; ^2^ Department of Food Nutrition and Health Biotechnology, Asia University, Taichung, Taiwan; ^3^ Graduate Institute of Health Industry Technology, Research Center for Food and Cosmetic Safety, and Research Center for Chinese Herbal Medicine, College of Human Ecology, Chang Gung University of Science and Technology, Taoyuan, Taiwan; ^4^ Department of Gastroenterology and Hepatology, Chang Gung Memorial Hospital, Taoyuan, Taiwan; ^5^ Department of Pharmacology, Yale University School of Medicine, New Haven, CT, United States; ^6^ Chinese Medicine Research Center, Department of Chinese Pharmaceutical Sciences and Chinese Medicine Resources, China Medical University, Taichung, Taiwan

**Keywords:** phytochemistry, historical source, herbal tea, qīng-căo-chá tea, field investigation, Taiwan traditional medicine, ethnobotany

## Abstract

Herbal tea, a beverage prepared from a variety of plant materials excluding the leaves of the tea plant *Camellia sinensis* (L.) Kuntze of the family Theaceae, for a long time, has been consumed by most Chinese people for preventive and/or therapeutic health care. Usually, it is brewed or prepared as a decoction of local plants in water. The qīng-căo-chá tea, a commercial herbal tea, is the most common among many differently formulated herbal teas in Taiwan. For hundreds of years, qīng-căo-chá tea has played an important role in the prevention and treatment of diseases associated with the environmental conditions in Taiwan. However, research studies in this field have been insufficient. The raw material formulas of qīng-căo-chá tea have always been passed down from “masters” to “apprentices.” Hence, there is no systematic collation or record, and, therefore, there is a need to assess and confirm the composition, safety, and effectiveness of the raw materials. This study aimed to document the uses of Taiwan's qīng-căo-chá tea through a semi-structured interview survey and investigate the background of traditional practitioners, tea compositions, and plant origins and uses. This will improve our understanding of the knowledge inherited by the practitioners and the theoretical basis of the medicinal uses of these teas. In our field investigation, we visited 86 shops and assessed 71 raw ingredients of qīng-căo-chá tea. A semi-structured questionnaire was used to conduct the interviews. During the interviews, in addition to written records, audio and video recordings were made, and photographs were taken with the permission of the interviewees. The qīng-căo-chá raw materials have long been used as herbal teas, although more research should clarify their efficacy and safety. Traditional sellers of qīng-căo-chá tea were mainly males, and most shops have been in operation for more than 71 years. Some of the raw materials were derived from multiple sources, including different plants, and were often mixed without any safety concerns. To our knowledge, this is the first systematic ethnobotanical study on qīng-căo-chá tea that assesses and confirms its herbal ingredients. Our study represents a reference for herbal teas in Taiwan that can be used by the public and regulatory agencies.

## Introduction

Herbal tea, a beverage prepared from a variety of plant materials excluding the leaves of the tea plant *Camellia sinensis* (L.) Kuntze of the family Theaceae, for a long time, has been consumed by most Chinese people for preventive and/or therapeutic health care (Han et al., 2013; Xiao et al., 2014). It is typically brewed or prepared as a decoction of local plants in water (Fu et al., 2018). Hence, in different regions, herbal teas have their distinct characteristics and are mainly influenced by local culture. In pan-Chinese areas, they have been used for more than 2000 years (Liu et al., 2013b; Han et al., 2013; Xiao et al., 2014). Interestingly, the use of herbal teas is often related to environmental conditions and the local climate. For example, herbal teas commonly used in the Lingnan area in China are also known as liáng chá. Due to the humid and sultry climate in this area, diseases related to “dampness syndrome” and “heat syndrome” occur frequently, and the local people experience “yin deficiency” as they are prone to excessive sweating. As a remedy, the people consume a local drink called liáng chá, which has the main functions of clearing heat, dispelling dampness, and nourishing yin (Tan et al., 2017). The effects of the ingredients of this tea beverage include clearing heat, detoxification, and relieving heatstroke (Li et al., 2017). Today, herbal teas have also been developed for use as drugs. In Guangzhou, an over-the-counter drug, “Guangdong Liáng Chá Granules,” was developed by Wanglaoji Herbal Tea. Multiple studies have suggested that the drug's potency in purging heat could be related to the antioxidative properties of the herbal ingredients (He et al., 2009). This example shows that herbal teas play an important role in disease prevention and treatment in Pan-Chinese areas.

Although Chinese herbal teas have a wide variety of applications, research articles remain insufficient internationally. Previous ethnobotanical studies related to liáng chá in the Lingnan area (Liu et al., 2013b) and Chaoshan area (Li et al., 2017) and three review article on Chinese herbal teas (Han et al., 2013; Xiao et al., 2014; Fu et al., 2018) provide valuable insights into this research field. Taiwan is an island in East Asia. According to the Executive Yuan's country profile, the population is composed of Han people (97%), aboriginals (2%), and others (1%). A survey of plant resources in Taiwan, published as the “2017 Taiwan Vascular Plant Red Data Book List,” includes a total of 5,188 wild vascular plant species, with one-quarter identified as “endemic species in Taiwan.” These statistics suggest that the different ethnic groups of Taiwan's population and the great variety of plant resources are contributing factors to the use of these herbal teas as health-promoting beverages in Taiwan.

There are many differently formulated herbal teas in Taiwan; among these, the qīng-căo-chá tea, a commercial herbal tea, is the most common. Qīng-căo-chá tea, also known as băi-căo-chá tea, is a regional beverage that originated through the accumulation of local knowledge. Its raw materials are mainly local wild or cultivated plants, which are collected and further processed using simple procedures (Qiu, 2004). Taiwan has a tropical and subtropical monsoon climate, and the weather in summer is often very humid. Hence, the main purpose of this traditional beverage is to relieve the physical discomfort under such circumstances. Its common effects are clearing heat and removing dampness, but it is also palatable, which increases the population's willingness to drink this tea in such a hot climate. Thus, qīng-căo-chá tea is one of the most important traditional beverages in Taiwan.

With the increasing awareness of health maintenance, more people are consuming natural health-promoting drinks. However, research studies on this aspect in Taiwan are insufficient. The formulas of raw materials for qīng-căo-chá tea have always been passed down verbally by tea shop owners, and there is no systematic collation or record. A book on prior related research (Qiu, 2004) has collated 24 formulas and 137 raw materials. Although this book focuses on qīng-căo-chá tea, the sample number of traditional users appears to be insufficient. Other publications include a master's thesis on the investigation of qīng-căo pharmacies (Jiang, 2005) and a government research project (CCMP93-RD-037), which investigated 86 shops and documented 47 commonly used raw materials for qīng-căo-chá tea. However, this work was not focused on qīng-căo-chá tea; instead, it only briefly mentioned this tea. There has been no systematic study on qīng-căo-chá tea varieties in Taiwan, indicating the need for further in-depth investigation and evaluation.

To record and better understand the characteristics of Taiwan's qīng-căo-chá tea, we conducted a survey of qīng-căo-chá teashops across Taiwan and carried out interviews using semi-structured questionnaires to assess the current situation and the raw materials of qīng-căo-chá tea used to date. We focused on identifying the unique cultural characteristics of Taiwan that facilitated the development of Taiwan's qīng-căo-chá tea.

## Materials and Methods

### Survey Area

**Figure 1:**
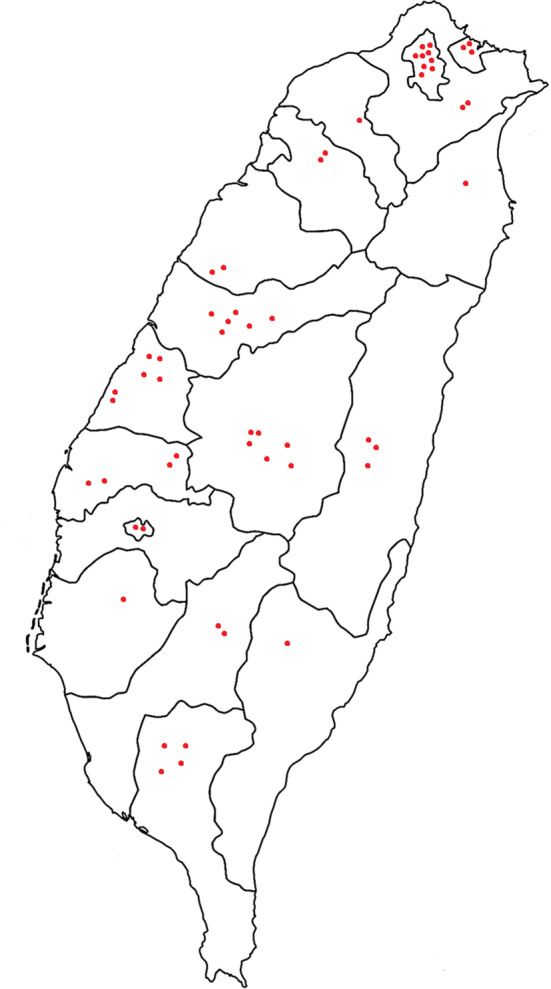
MAP showing the geographical distribution of the qīng-căo-chá tea shops in Taiwan investigated in this study.

Taiwan is an island located in East Asia (21°45′-25°56′N, 119°18′-124°34′E), spanning the Tropic of Cancer, with an area of 36,197 km^2^. It has a tropical monsoon and subtropical monsoon climate. The shops visited during this study were randomly distributed across Taiwan (**Figure 1**). The study was performed from May 2018 to November 2018 and was reviewed and approved by the Regional Research Ethics Center in Taiwan (CRREC-107-019).

### Interviews and Data Collection

In this study, we used a semi-structured questionnaire to conduct the interviews. The target subjects were shops that sold self-prepared qīng-căo-chá tea according to information found on the internet or provided by various local medicinal plant-related organizations. The studies involving human participants were reviewed and approved by Regional Research Ethics Center (CRREC-107-019). The patients/participants provided their written informed consent to participate in this study. Among a total of 86 visited shops, 42 participated in a complete interview, whereas 27 refused to participate in the interview, 13 only provided public information on the formula used in the shop, and 4 did not sell qīng-căo-chá tea. During the interview, in addition to written records, audio and video recordings were made, and photographs were taken with the permission of the interviewees. The origins of all medicinal materials (samples retained at the Department of Pharmacy, China Medical University) were identified by Dr. Shyh-Shyun Huang (Associate Professor, School of Pharmacy, China Medical University). The herbarium voucher is placed in the department of Chinese Pharmaceutical Sciences and Chinese Medicine Resources, China medical university.

The information obtained in the interview consisted of two parts, the basic information, including the age and gender of the interviewees as well as the shop location, and the answers to six semi-structured questions, as follows: (1) how was the traditional knowledge obtained; (2) for how long has the shop been operating; (3) whether there are commodities other than tea sold; (4) the composition of qīng-căo-chá tea; (5) the effects of this qīng-căo-chá tea; and (6) other relevant information.

### Data Analysis

All assessed plant data are presented in Appendix A, **Table A.1**, which contains (1) scientific names, (2) family names, (3) parts used, (4) name of medicinal materials, (5) number of times the surveyed qīng-căo-chá tea shops used them, (6) use value (UV), (7) comparison to items investigated in nearby areas (Liu et al., 2013b; Lin, 2014; Li et al., 2017), and (8) traditional efficacy information.

The scientific names (1) and plant family names (2) in **Table A.1** are based on “The Plant List (www.theplantlist.org).” The scientific name in “[*genus species*]” means that the scientific name often appears in pharmacopoeia and botanical records (the “Taiwan Herbal Pharmacopoeia,” 3^rd^ Edition (Editing Working Group of Ministry of Health and Welfare, 2018), the Enumeratio Plantarum Formosanarum, 2^nd^ Edition (Editorial Committee of Flora of Taiwan, 2003), and the Taiwan Biodiversity Information Facility (http://taibif.tw/)); columns (3) and (4) of **Table A.1** present the data from this study; column (5) shows the number of qīng-căo-chá tea shops that used the sample in the survey; the UV in column (6) were calculated using the following formula: UV (%) = frequency/sample numbers × 100 (Yemele et al., 2015); the information in column (8) on the traditional efficacy is based on the “List of Medicinal Plant Resources in Taiwan” (Committee on Chinese Medicine and Pharmacy, 2003) and the Second Edition of the “Illustrated Handbook of Commonly Used Medicinal Plants in Taiwan” (Committee on Chinese Medicine and Pharmacy, 2011–2012).

To assess the current pharmacological research status of these raw materials, we identified 26 raw materials with UV greater than 5 (Appendix B) and searched the PubMed database (https://www.ncbi.nlm.nih.gov/pubmed/) for the pharmacological studies conducted on those materials.

### DPPH (1,1-Diphenyl-2-Picrylhydrazyl) Free Radical Scavenging Analysis

Previous studies indicated that the action of clearing heat appears to be related to antioxidative effects *in vivo* (He et al., 2009). To further assess the activity profile of the qīng-căo-chá tea raw materials, we tested the raw plant materials with UV greater than 5 for antioxidant activity. However, if the material was included in the “Taiwan Herbal Pharmacopoeia,” 3^rd^ Edition (Editing Working Group of Ministry of Health and Welfare, 2018), it was not tested in this assay. The purple-blue 1,1-diphenyl-2-picrylhydrazyl (DPPH) powder consists of stable free-radical molecules, which have a strong absorbance at 517 nm in solution. When DPPH reacts with a hydrogen-donating antioxidant, it results in discoloration and a drop in absorption at 517 nm; the lower the absorption value, the stronger the hydrogen-donor ability of the antioxidant (AH; i.e., the stronger the antioxidant's ability to scavenge free radicals). The reaction for reducing the DPPH radical (DPPH•) by an antioxidant (AH) is as follows:

DPPH•(violet) + AH → DPPH : H(decolorized) + A•

We prepared 95% alcohol extracts of the herbal materials (250 µg/ml) as the test samples. An equal concentration of butylated hydroxytoluene (BHT) was used as the control. The assay was performed as follows: 20 µL of a prepared sample was added to 80 µL Tris-HCl (pH=7.4) and mixed with 100 µL DPPH (500 µM) (Sigma-Aldrich). The reaction mixture was protected from light for 30 min, and the absorption was measured at 517 nm using a microplate reader (SpectraMax iD3 Multi-Mode Microplate Reader; Molecular Devices, San Jose, CA, USA). The raw DPPH scavenging rates of the extracts were calculated and converted to the relative DPPH scavenging rates (%) of BHT using the scavenging rate of BHT in the positive control group as the denominator. The DPPH scavenging rates of individual extracts were calculated using the following formula:

$$\left(1-\frac{Abs\left(sample\right)}{Abs\left(control\right)}\right)\times 100\%$$

## Results and Discussion

### Information on Interviewees

In this study, we interviewed 42 traditional sellers of qīng-căo-chá tea (**Table 1**). According to the data presented in **Table 2**, traditional sellers of qīng-căo-chá tea were mainly males (80.95%). This might be related to this society's traditional preference for males over females and the fact that the formulas are only passed on to males. Moreover, because boiling tea is a labor-intensive job, males are thought to have more physical strength to engage in this occupation.

**Table 1:** The information of Qīng-căo-chá tea store.

Store code	Location	Description
TC1	Taichung City, Taiwan	The stores are located in “Chinese herb street,” Taichung. The “Chinese herb street” is the distribution center of Qīng-căo-chá tea stores in the central Taiwan since ancient times.
TC2	Taichung City, Taiwan	
TC3	Taichung City, Taiwan	
TC4	Taichung City, Taiwan	
TC5	Taichung City, Taiwan	
TC6	Taichung City, Taiwan	A hundred years old Qīng-căo-chá tea store
TP1	Taipei City, Taiwan	A hundred years old Qīng-căo-chá tea store in the “Qingcao Street” of Longshan Temple
TP2	Taipei City, Taiwan	The “Qingcao Street” of Longshan Temple is the most important distribution center of Northern Taiwan. The vicinity of Longshan Temple is one of the earliest development areas in Taiwan, with a long history.
TP3	Taipei City, Taiwan	
TP4	Taipei City, Taiwan	
TP5	Taipei City, Taiwan	
TP6	Taipei City, Taiwan	
TP7	Taipei City, Taiwan	
TP8	Taipei City, Taiwan	
TP9	Taipei City, Taiwan	
TT1	Taitung County, Taiwan	Taitung County is one of the cultivated place of the raw materials in the eastern Taiwan. The store is the wholesaler.
TN1	Tainan City, Taiwan	The Qīng-căo-chá tea store is in ancient traditional bazaar and near temple
YL1	Yilan County, Taiwan	The stores are located in the distribution center in the eastern Taiwan since ancient times.
HL1	Hualien County, Taiwan	Hualien County is one of the important cultivated place of the raw materials in the eastern Taiwan.
HL2	Hualien County, Taiwan	
HL3	Hualien County, Taiwan	
NT1	Nantou County	
NT2	Nantou County	
NT3	Nantou County	
NT4	Nantou County	
NT5	Nantou County	
NT6	Nantou County	
PT1	Pingtung county	Pingtung county is one of the cultivated place of the raw materials in the southern Taiwan.
PT2	Pingtung county	
PT3	Pingtung county	
PT4	Pingtung county	
ML1	Miaoli county	
ML2	Miaoli county	
TY1	Taoyuan	
KS1	Kaohsiung	The Sunfong Palace is the most famous distribution center of Qīng-căo-chá tea stores in the southern Taiwan since ancient times.
KS2	Kaohsiung	
KL1	Keelung	
KL2	Keelung	
KL3	Keelung	
YL1	Yunlin County	Yunlin County is the most important cultivated place of the raw materials in Taiwan. More than 200 kinds of medicinal plants are planted. 20 kinds are planted in large quantities
YL2	Yunlin County	
YL3	Yunlin County	
YL4	Yunlin County	
NT1	New Taipei City, Taiwan	
NT2	New Taipei City, Taiwan	
HC1	New Taipei City, Taiwan	
HC2	New Taipei City, Taiwan	
CY1	Chiayi County, Taiwan	
CY2	Chiayi County, Taiwan	
CH1	Changhua County, Taiwan	One of the earliest development areas in Taiwan with a long history
CH2	Changhua County, Taiwan	
CH3	Changhua County, Taiwan	
CH4	Changhua County, Taiwan	
CH5	Changhua County, Taiwan	
CH6	Changhua County, Taiwan	

**Table 2:** Statistics on interviewees of qīng-căo-chá tea (N=42).

Item	Description	Percentage
Gender	Male	80.95%
	Female	19.05%
Age (years)	21-30	4.76%
	31-40	21.43%
	41-50	21.43%
	51-60	26.19%
	61-70	16.67%
	Over 71	9.52%
Source of knowledge	Inherited through family line	80.95%
	Acquired through mentorship	11.90%
	Personal experience	7.14%
Shop history (years)	Less than 10	4.76%
	11-20	11.90%
	21-30	16.67%
	31-40	11.90%
	41-50	4.76%
	51-60	11.90%
	61-70	7.14%
	Over 71	30.95%
Other items sold	Tea beverages only	19.04%
	Tea beverages and dried raw materials	40.48%
	Tea beverages and dried and fresh raw materials	40.48%

Qīng-căo-chá tea sellers were mainly between 51 and 60 years of age (26.19%). We also met many sellers over 60 years of age who were still operating their business. Thus, we assumed that the industry is aging and lacks the input of young people.

Remarkably, formulas passed on within families accounted for the highest proportion (80.95%) among the knowledge sources, thereby revealing the dominant role of traditions in this industry. The information on these formulas is often kept within families, which indicates the conservative nature of this business.

### Shop Information

According to **Table 2**, shops that have been operating for more than 71 years (30.95%) accounted for the highest proportion of sellers. Most stores were passed on from generation to generation, corroborating our observation that family inheritance is the main source of knowledge. It is presumed that operators of traditional beverage shops are very conservative, and almost all inherit their business *via* a direct line of succession. Therefore, when the use of these traditional teas decreases, the heritage of related knowledge will gradually disappear.

Besides selling tea, most shops (80.96%) sold related fresh or dry raw plant materials. This finding shows that traditional tea sellers do not rely solely on tea beverages as their main source of business. In addition, they retail some raw materials or traditional Chinese medicines according to local uses and traditions. Some sellers also served as local folk medicine practitioners. However, with constant development and progress in the medical field, many shops have begun selling traditional drinks only or have changed the traditional qīng-căo-chá tea into a new type of hand-shake drink to continue this traditional culture.

### Plants Used

**Figure 2:**
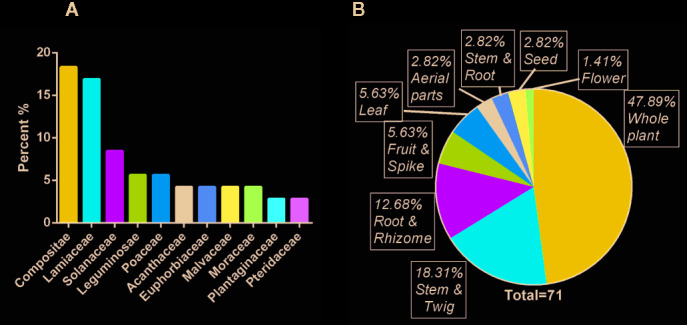
General characteristics of raw materials used for preparing qīng-căo-chá tea. **(A)** Proportions of plant families in raw materials; **(B)** plant parts used as raw materials.

**Figure 3:**
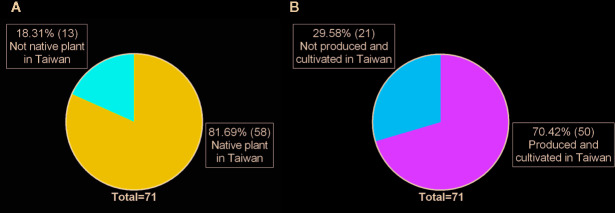
The distribution **(A)** and the proportion of cultivation **(B)** of qīng-căo-chá tea raw materials in Taiwan.

This study identified 71 raw materials from 69 plants belonging to 31 families and 65 genera (**Appendix A**). The plant species with the highest UV was *Platostoma palustre* (Blume) A.J.Paton (72.73%), followed by *Bidens pilosa* L. (49.09%) and *Pteris multifida* Poir. (43.64%). The most commonly used plants belonged to the family Compositae (18.84%), followed by the family Lamiaceae (15.94%) (**Figure 2A**). The raw materials were mainly prepared from whole plants (47.89%), followed by stems and twigs (18.31%) (**Figure 2B**). It is worth noting that these plants are a high proportion of native Taiwan species and are grown in Taiwan (**Figure 3**).

We identified *P. palustre* (Blume) A.J.Paton as the most commonly used plant. The traditional applications of *P. palustre* (Blume) A.J.Paton are to clear heat, quench thirst, and provide relief from summer heat (Committee on Chinese Medicine and Pharmacy, 2003). The pharmacological properties studied so far include its antidiabetic (Yuris et al., 2018) and antioxidative activities (Tang et al., 2017). Furthermore, the frequency of its use was ranked 9^th^ in a previous study (Qiu, 2004), but 1^st^ in this study. This might be because *P. palustre* (Blume) A.J.Paton has been actively promoted in Hsinchu County and Miaoli County in Taiwan in recent years, especially in the Kuanhsi Town of Hsinchu County, where *P. palustre* (Blume) A.J.Paton is widely cultivated. The local farmers' association has also guided many *P. palustre* (Blume) A.J.Paton processing plants and created several products for promoting this commodity (such as the *P. palustre* (Blume) A.J.Paton tea, *P. palustre* (Blume) A.J.Paton jelly, and *P. palustre* (Blume) A.J.Paton chicken stewing bag, etc.). In addition, the use of *P. palustre* (Blume) A.J.Paton has a long history in Taiwan, and its flavor is generally accepted by the local population. Beverages with *P. palustre* (Blume) A.J.Paton have a deep color, aroma, and an “ancient taste”; and therefore, this source plant is a suitable raw material for traditional qīng-căo-chá tea. Most shop sellers mentioned that *P. palustre* (Blume) A.J.Paton is inexpensive, and the longer it is stored, the better it tastes. Overall, its taste is easily accepted by the public. Interestingly, among the liáng chá raw materials used in the Guangdong area, *P. palustre* (Blume) A.J.Paton, also known as liáng-fen-căo, is an important herbal tea ingredient because of its heat-clearing effect. It is also the main constituent of guiling jelly (Guīlínggāo) and has been widely used in herbal teas in different regions due to its medicinal properties. Further research and development should be focused on this plant due to its wide application in food and its pharmacological effects.

Besides *P. palustre* (Blume) A.J.Paton, most of the raw materials are plants that are commonly found in the wild in Taiwan, which corresponds with the definition that raw materials for herbal teas are mainly “derived from local plants.” However, there are still some raw materials that are not produced in Taiwan, such as *Glycyrrhiza uralensis* Fisch. These raw materials are traditional Chinese medicinal materials commonly used in Taiwan and are in conformance with the principle of “using local materials.” Therefore, the commonly used raw materials for herbal teas are not invariable; instead, they vary between different environments, local populations, and medical applications. However, raw materials that are easily accessible are given priority during selection.

In our field investigation, we found that the traditional shops used different raw materials depending on their environment. Specifically, the use of *Mentha arvensis* L. had the most significant regional differences. *M. arvensis* L. is a plant from the family Lamiaceae. Because its volatile oil generates a cooling sensation, it is a critical raw material in qīng-căo-chá tea. We found that shops in central Taiwan (Taichung City, Nantou County, and Changhua County, etc.) did not favor the use *M. arvensis* L., whereas large amounts were used in counties and cities in southern Taiwan, south of the Tropic of Cancer. Such differences may be related to climate and local hobbies. In the hot climate of southern Taiwan, the use of *M. arvensis* L. can immediately generate a cooling sensation in individuals. However, in the mountainous areas of the central region with a mild climate, this effect may not be of importance in a local tea formula.

The largest proportion of plants belonged to the family Compositae with a wide variety of species commonly found in Taiwan. According to the records in Volume 6 of the 2^nd^ Edition of *Enumeratio Plantarum Formosanarum*, more than 6,200 species of vascular plants have been identified in Taiwan to date. The family Compositae, with 85 genera and 242 species, represents the third-largest vascular plant family in Taiwan. This family also includes a large number of naturalized and invasive plants from around the world (Zhong et al., 2008). Thus, plants of the family Compositae are easily accessible in the wild in Taiwan. Because the raw materials of qīng-căo-chá tea are locally obtained, plants of this family are a favorite raw material source for qīng-căo-chá tea producers.

In addition, in this study, we found that some raw materials used for qīng-căo-chá tea were prepared from different plant species (**Table 3**) that were often mixed. For example, *Artemisia capillaris* Thunb. and *Origanum vulgare* L. were both used as source plants of the oriental wormwood herb, and both *Oldenlandia diffusa* (Willd.) Roxb. and *Oldenlandia corymbosa* L. were the source plants of the spreading hedyotis herb without differentiation. This use habit is a common source of confusion in Taiwan's medicinal material market. These plants are still being mixed up, probably due to their similar appearance or common name, and no safety concern has been reported regarding their use. The confusion of some closely related species appears to be a major feature in the use of medicinal materials in Taiwan.

**Table 3:** Common raw materials obtained from multiple sources.

Name of medicinal material	Original material mentioned in Pharmacopoeia or official literature	Material with Taiwanese characteristics
Oriental wormwood herb	*Artemisia capillaris* Thunb.	*Origanum vulgare* L.
Astragalus root	*Astragalus propinquus* Schischkin	*Hedysarum polybotrys* Hand.-Mazz.
Lalang grass rhizome	*Imperata cylindrica* (L.) Raeusch.	*Pennisetum flaccidum* Griseb.
Spreading hedyotis herb	*Oldenlandia diffusa* (Willd.) Roxb.	*Oldenlandia corymbosa* L.
Mongolian dandelion herb	*Taraxacum campylodes* G.E.Haglund	*Ixeris chinensis* (Thunb.) Nakai

### Efficacy

Qīng-căo-chá tea is sold as food, and its curative effect cannot be claimed according to law. During the survey, store owners stated that the traditional uses of qīng-căo-chá tea are cooling, quenching of thirst, promotion of fluid production, and reduction of internal heat. Therefore, this study further assessed the traditional efficacy of plant materials and the results from modern research.

The most common descriptions of the efficacy of traditional drugs are “nature” and “flavor.” To quote Shen Nong's “Materia Medica (*Shennong Ben Cao Jing*),” “one medicinal material has one nature and one flavor.” The description of nature and flavor is a historical method to describe the efficacy of medicinal materials.

**Figure 4:**
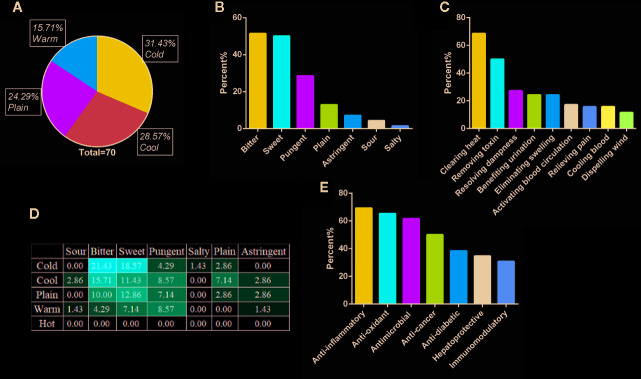
Properties of qīng-căo-chá tea raw materials. **(A)** Traditional natures of raw materials; **(B)** flavors of raw materials; **(C)** traditional effects of raw materials; **(D)** proportions (%) of flavors among the traditional natures of raw materials; and **(E)** modern research efficacy of qīng-căo-chá tea raw materials.

In traditional descriptions, the “four natures,” also called the “four properties,” are divided into five different degrees, “hot,” “warm,” “plain,” “cool,” and “cold,” to describe the efficacy of traditional Chinese medicinal materials. A literature analysis showed that a total of 70 plant materials had been recorded previously. The most common characteristics of plant materials were cold (31.43%) and cool (28.57%) (**Figure 4A**). Based on this finding, we assumed that the “heat-clearing” effect of qīng-căo-chá tea should be related to its medicinal property, which aims to improve the “heat syndrome” symptoms (fever; flushed face; dysphoria and feverish sensation in chest, palms, and soles; scanty dark urine; and dry bound stool; etc.) in traditional diagnosis. Interestingly, qīng-căo-chá tea also contains a small proportion of warm raw materials (15.71%). According to interviews and investigations, the warm raw materials are typically used to “moderate the drug properties.” The concept of balance between cold and heat in traditional efficacy descriptions and the concept of balance between yin and yang in traditional Chinese medicine are embodied in the compatibility of the raw materials used in qīng-căo-chá tea.

Traditional efficacy descriptions identify five common “flavors,” i.e., “sour,” “bitter,” “sweet,” “acrid (pungent),” and “salty,” plus “plain,” and “astringent.” These categories represent three levels of meaning: (1) actual taste, (2) chemical composition changes, and (3) generalization of efficacy. Therefore, the description of “flavors” is a specific assessment of traditional efficacy. The classification of the flavors of qīng-căo-chá tea raw materials showed that most of them were either bitter (51.43%) or sweet (50.00%) (**Figure 4B**). It is presumed that the qīng-căo-chá tea flavors are related to its efficacy and taste. Traditional practitioners in Taiwan have the perception that bitter ingredients protect the liver and reduce liver fire (here, the actual liver is referred to and not the liver described as the viscera of traditional Chinese medicine). Hence, the bitter flavor is related to the choices made to achieve efficacy. Interestingly, traditional Chinese medicine suggests that bitter medicinal materials enter the heart meridian (viscera and heart meridians of traditional Chinese medicine) and have the effects of clearing heat and eliminating dampness. Therefore, the perception of bitter among traditional Taiwanese practitioners differs from the traditional Chinese medicine theory. In addition, sugar or “sweet flavor” raw materials are added to improve the palatability of the tea. “Sweet flavor” also has the traditional meaning of “nourishing, harmonizing various drugs, and regulating the middle warmer,” which can ease the cold nature of the bitter flavor. Therefore, the sweet flavor is also related to choices for efficacy and taste. Moreover, many people do not consider sugary teas as healthy. Therefore, it is very important to adjust the palatability with sweet raw materials.

According to our analysis (**Figure 4D**), the raw materials of qīng-căo-chá tea were mainly “bitter and cold,” “bitter and cool,” and “sweet and cold.” The traditional effects of most raw materials were “clearing heat, removing toxicity, and removing dampness” (**Figure 4C**), which aligned with the efficacy results of qīng-căo-chá tea discussed in the survey. Its main effects of clearing heat, removing toxicity, and removing dampness might also be related to the humid summer weather in Taiwan. It was mentioned several times in the interviews that qīng-căo-chá tea is a popular refreshment in the summer. Thus, qīng-căo-chá tea alleviates the problems of the dampness syndrome and heat syndrome in the summer, similar to the Guangdong herbal tea mentioned by Tan et al. (2017) but based on different effects.

Twenty-six raw materials with UV greater than 5 were assessed for modern pharmacological effects based on recent research results (**Figure 4E**, **Table B.1**). Anti-inflammatory and antioxidative effects were the most common properties, corroborating the finding of a previous research report that reducing heat is related to oxidative stress relief *in vivo* (He et al., 2009).

### Comparison With Neighboring Areas

According to the country profile provided by the Executive Yuan, Taiwan's population is mostly Han. During the Qing dynasty, most people immigrated from Quanzhou (45%), Zhangzhou (35%), and Chaozhou (16%), which are located in the area between the Fujian and the Guangdong province of modern China (Zhou, 2016). This area also has locally popular herbal drinks, including the Guangdong herbal tea. Therefore, to investigate whether the geographical location and cultural background affected the selection and use of raw materials in Taiwan, this study compared the materials used in the Lingnan area (Liu et al., 2013b), Chaoshan area (Li et al., 2017), and Fujian area (Lin, 2014) with the survey results of our study.

**Figure 5:**
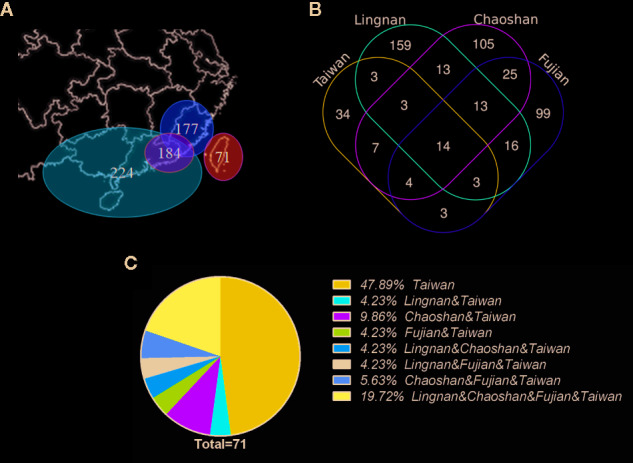
Diversity of raw materials used for preparing herbal teas in Taiwan and neighboring areas. **(A)** Map of geographic areas compared in this study (numbers of raw materials per area are shown); **(B)** Venn diagram derived from comparisons of raw materials used in different geographic areas (numbers of raw materials are shown); **(C)** proportions of overlapping raw material use between Taiwan and neighboring areas.

Remarkably, 34 (47.89%) of the 71 raw materials are only used in Taiwan (**Figure 5**). This finding indicates that Taiwan has its own unique selection of qīng-căo-chá tea raw materials after nearly 400 years of independent development.

One of the common materials, *Glossocardia bidens* (Retz.) Veldkamp, is a main product in the Penghu area. This material has Taiwan characteristics, and its selection is related to the location on the island. It has the effects of clearing heat and removing toxicity, promoting diuresis, and reducing swelling (Committee on Chinese Medicine and Pharmacy, 2003). In recent years, many pharmacological studies have confirmed its immunomodulatory (Wu et al., 2005), anti-inflammatory, and other beneficial effects (Ha et al., 2006). Because of its special flavor and efficacy, its use in herbal beverage raw materials has gradually increased.

We identified 14 species (19.72%) that are used in Lingnan, Fujian, Chaoshan, and Taiwan. It is assumed that these plants grow in these regions due to the similar geographical environment and climate. Moreover, these plants are used to prevent and treat common diseases in similar geographical environments.

### Results of Antioxidant Activity Analysis

Previous studies discussed that the efficacy of herbal tea in reducing heat is related to minimizing the effects of oxidative stress *in vivo* (He et al., 2009). In this study, we evaluated the DPPH antioxidant activity of plant raw materials with an UV greater than 5 to determine the effect of qīng-căo-chá tea raw materials. However, if the raw materials were included in “Taiwan Herbal Pharmacopoeia,” they were not tested in this experiment.

**Figure 6:**
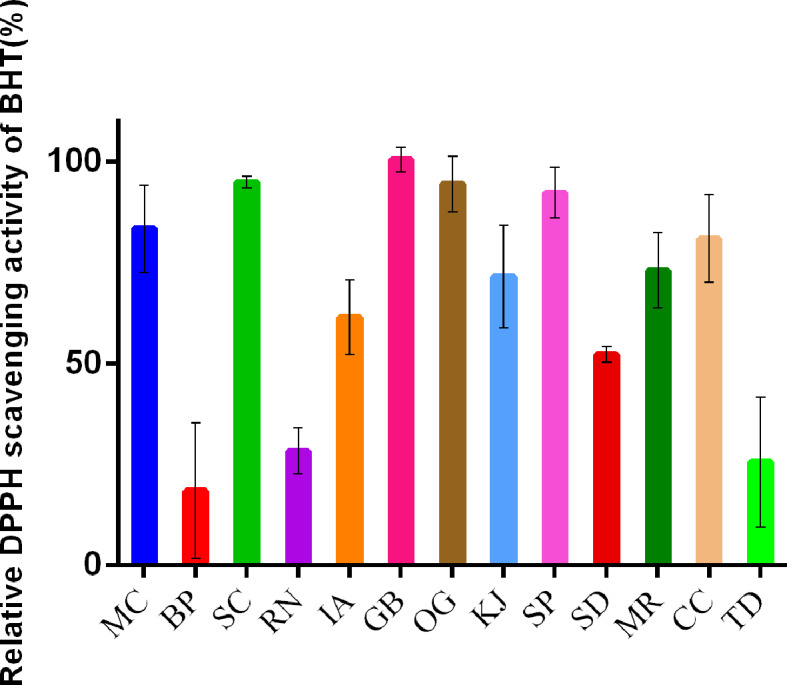
Comparison of antioxidant activities of alcohol extracts from raw materials of qīng-căo-chá tea with UV>5. The raw materials are presented in descending order using the raw material UV. Extracts were tested at a concentration of 250 µg/ml, and the scavenging rate of each raw material was converted into the relative DPPH scavenging rate (%) of BHT using the scavenging rate of the positive control with BHT as the denominator. Values are expressed as the mean ± standard deviation (n=4). Plant name codes: PP, *Platostoma palustre* (Blume) A.J.Paton; BP, *Bidens pilosa* L.; SC, *Sphagneticola calendulacea* (L.) Pruski; RN, *Rhinacanthus nasutus* (L.) Kurz; IA, *Ilex asprella* (Hook. & Arn.) Champ ex Benth.; GB, *Glossocardia bidens* (Retz.) Veldkamp; OG, *Ocimum gratissimum* L.; KJ, *Kadsura japonica* (L.) Dunal; SP, *Salvia plebeia* R. Br.; SD, *Scoparia dulcis* L.; MR, *Mallotus repandus* (Willd.) Muell.-Arg.; CC, *Clerodendrum cyrtophyllum* Turcz.; and TD, *Tithonia diversifolia* (Hemsl.) A. Gray.

This experiment indicated that the DPPH radical scavenging capacity of alcohol extracts from most raw materials is greater than 50% of the DPPH radical scavenging capacity of BHT (**Figure 6**). Extracts without activity in this assay were made from *Bidens pilosa* (BP), *Rhinacanthus nasutus* (RN), and *Tithonia diversifolia* (TD). Overall, our results corroborated previous reports that the efficacy of liáng chá in reducing heat was related to antioxidative effects *in vivo* and that qīng-căo-chá tea exhibited health-restoring efficacy in clearing heat due to the antioxidative activity of the raw material constituents.

## Conclusions

In historical and modern times, herbal teas have benefited human health and quality of life. Qīng-căo-chá tea is an important traditional beverage in Taiwan that was developed over a long historical period when convenient access to traditional local materials was critical. Today, further commercial changes have taken place due to the development of modern logistics. However, with changes in society, the traditional use of local materials has become a gradually disappearing culture, and finding ways and innovations to continue this tradition in this era is a major issue. Furthermore, managing the safety and efficacy of different raw materials is also critical for their continued use. To our knowledge, this study is the first to explore and scientifically sort the basic characteristics and uses of Taiwan qīng-căo-chá tea. Further research is needed to clarify its efficacy and safety, which will promote better use of qīng-căo-chá tea.

## Data Availability Statement

All datasets generated for this study are included in the article/**Supplementary Material**.

## Author Contributions

S-SH, T-YC, J-SD, L-HP, Y-CC, and JC designed the study. S-SH, T-YC, J-SD, and JC conducted the field work. S-SH, T-YC, L-HP, and JC performed the data analysis. S-SH, T-YC, Y-CC, and JC wrote the manuscript. All authors contributed to the article and approved the submitted version.

## Funding

We would like to express our gratitude to the Ministry of Science and Technology for providing the funding for this paper through the following projects: MOST 107-2320-B-039-030-MY3 and MOST 107-2320-B-039-063. This work was also supported by a grant from the China Medical University (CMU106-N-24) and a grant for the Chang Gung Medical Research Program (CMRPF1D0123) from the Chang Gung Memorial Hospital. Further, this study was supported by the China Medical University under the Higher Education Sprout Project and Teaching Practice Research Program (1077170A), Ministry of Education, Taiwan.

## Conflict of Interest

The authors declare that the research was conducted in the absence of any commercial or financial relationships that could be construed as a potential conflict of interest.

## Abbreviations

BHT, butylated hydroxytoluene; UV, use value.
