# Supplementary material for: An Ethnobotanical Study on Qīng-Căo-Chá Tea in Taiwan
Source: Front Pharmacol. 2020 Jun 25;11:931. doi: 10.3389/fphar.2020.00931 (PMC7329985; doi:10.3389/fphar.2020.00931)
Supplement: Supplementary file 1 [file DataSheet_1.pdf]

Appendices

Appendix A

Table A.1

General information on raw materials of qīng-cǎo-chá tea and their traditional uses (N=55).

No.	Scientific name	Family	Parts used	Local name	Frequency	UV (%)	Additional information				Traditional uses
							The first record in the literature before the Qing Dynasty	Comparative data	Native plant in Taiwan	Produced and cultivated in Taiwan	
1.	*Abrus pulchellus* subsp. *cantoniensis* (Hance) Verdc.	Leguminosae	Whole plant	Jī gǔcǎo (雞骨草)	1	1.82	—	AB	N	N	Sweet and bland in taste and cool in nature. Effects: clearing heat, promoting diuresis, soothing liver, and relieving pain. Used for treating hepatitis, liver cirrhosis, and stomachache.

2.	*Acalypha australis* L.	Euphorbiaceae	Whole plant	Jīnxièliú (金榭榴)	1	1.82	Zhíwùmíngshítúkǎo 《植物名實圖考》	BC	Y	Y	Bitter and astringent in taste and cool in nature. Effects: clearing heat and toxicity, inducing diuresis, relieving dysentery, killing parasites, and stopping bleeding. Used for treating bacillary dysentery, intestinal diarrhea, hematochezia, hematemesis, cough, malnutrition, metrorrhagia, abdominal distension, dermatitis, eczema, and traumatic hemorrhage.
3.	*Ajuga nipponensis* Makino	Lamiaceae	Whole plant	Bái mǎwú gong (白馬蜈蚣)	1	1.82	Běn cǎotújīng《本草圖經》		Y	Y	Bitter in taste and cold in nature. Effects: anti-inflammatory

	effect, blood cooling, and bone strengthening. Used for treating abscess, wind-heat cough and asthma (phlegm), tonsillitis, mumps, acute cholecystitis, hepatitis, dysentery, syphilis, hemorrhoids, tumors, epistaxis, toothache, conjunctival congestion and swelling, hematochezia, proteinuria, blood stasis and swelling, postpartum blood stasis, blood qi pain in women, and alopecia areata in children.

4.	*Alternanthera sessilis* (L.) R.Br. ex DC.	Amaranthaceae	Whole plant	Hóngtiánwū (紅田烏)	2	3.64	Jiùhuāngběncǎo《救荒本草》	BC	Y	Y	Bitter in taste and cool in nature. Effects: clearing heat, inducing diuresis, and detoxifying. Used for treating cough, hematemesis, intestinal bleeding, gonorrhea, kidney disease, and dysentery.
5.	*Artemisia capillaris* Thunb.*	Compositae	Whole plant	Yīn chén (茵陳)	14	25.45	Shén nóng běn cǎo jīng《神農本草經》		Y	N	Bitter and acrid in taste and cool in nature. Clearing heat and promoting diuresis, promoting gallbladder, and relieving jaundice. Used for treating jaundice, cholecystitis, damp-heat in bladder, dysuria, wind itch, sore, and scabies.

6.	*Asparagus cochinchinensis* (Lour.) Merr.	Asparagaceae [Liliaceae]	Root	Tiān mén dōng (天門冬)	1	1.82	Shén nóng běn cǎo jīng《神農本草經》	ABC	Y	N	Sweet in taste and bitter and cold in nature. Effects: nourishing yin, promoting fluid production, moistening lung, clearing heart fire, moistening dryness, clearing lung heat, and reducing fire. Used for treating yin deficiency, fever, cough, vomiting blood, lung abscess, and sore throat.
7.	*Astragalus propinquus* Schischkin* [*Astragalus membranaceus* (Fisch.) Bunge var. *mongholicus* (Bunge) P.K.Hsiao]	Leguminosae	Root	Huángqí (黃耆)	1	1.82	Shén nóng běn cǎo jīng《神農本草經》		N	N	Sweet in taste and warm in nature. Effects: invigorating qi and consolidating exterior, promoting diuresis, promoting detoxification, expelling pus, and promoting granulation. Used for treating

											fatigue from internal injury, diarrhea from spleen deficiency, cough, rectocele, hematemesis, hematochezia, metrorrhagia, spontaneous perspiration, night sweat, edema, and arthralgia.
8.	*Bidens pilosa* L. [*Bidens pilosa* L. var. *radiata* Sch. Bip.]	Compositae	Whole plant	Xián fēng cǎo (咸豐草)	27	49.09	—	B	Y	Y	Sweet and slightly bitter in taste, and mild in nature. Effects: clearing heat and toxicity, promoting diuresis, and eliminating jaundice. Used for treating common cold and fever, rheumatic arthralgia, damp-heat jaundice, carbuncle, swelling, sore, and furuncle.

9.	*Boehmeria nivea* (L.) Gaudich. [*Boehmeria nivea* (L.) Gaudich. var. *tenacissima* (Gaudich.) Miq.]	Urticaceae	Stem	Shānzhù má (山苎麻)	2	3.64	—	Y	Y	Sweet in taste and cold in nature. Anti-inflammatory and antipyretic. Used for treating hepatitis.
10.	*Bombax ceiba* L. [*Bombax malabaricum* DC.]	Malvaceae [Bombacaceae]	Root	Mù miángēn (木棉根)	2	3.64	—	Y	Y	Acrid in taste and neutral in nature. Effects: dispelling wind and eliminating dampness, cooling and detoxifying, dissipating blood stagnation, and relieving pain. Used for treating hepatitis, rheumatic arthralgia, gastric ulcer, dysentery, postpartum edema, scrofula, and traumatic injury.

11.	*Canarium album* (Lour.) DC.	Burseraceae	Root	Gǎn lǎn gēn (橄欖根)	1	1.82	—	C	Y	Y	Bland in taste and neutral in nature. Effects: clearing pharynx, detoxifying, and improving joints. Used for treating sore throat, beriberi, and muscle and bone pain.
12.	*Centella asiatica* (L.) Urb.	Apiaceae	Whole plant	Hán ké cǎo (含殼草)	2	3.64	Shén nóng běn cǎo jīng《神農本草經》	ABC	Y	Y	Bitter and acrid in taste, and cold in nature, with minor toxicity. Effects: anti-inflammatory effect, detoxifying, cooling blood, promoting fluid production, clearing heat, and promoting diuresis. Used for treating infectious hepatitis, measles, common cold, tonsillitis, pharyngolaryngitis, bronchitis,

										urinary tract infection, calculus, intestinal lavage, arsenic, and mushroom poisoning.
13.	*Chrysanthemum morifolium* Ramat.	Compositae	flower	Jú huā (菊花)	2	3.64	Shén nóng běn cǎo jīng《神農本草經》	ABC	N	Y (Miaoli County)	Sweet and bitter in taste and and in nature. Effects: dispelling wind and clearing heat, calming liver, improving eyesight, detoxifying, and promoting detumescence. Used for treating headache, vertigo, conjunctival congestion, and swelling.
14.	*Citrus medica* L. [*Citrus medica* L. var. *sarcodactylis* Swingle]	Rutaceae	Root	Fó shǒu gēn (佛手根)	1	1.82	—		N	Y	Acrid and bitter in taste and bland in nature. Effects: regulating qi and eliminating phlegm. Used for treating liver

											and stomach qi pain, spleen enlargement, and epilepsy.
15.	*Clerodendrum cyrtophyllum* Turcz.	Lamiaceae [Verbenaceae]	Stem & Root	Guān yīn chuàn (觀音串)	3	5.45	Míng yī bié lù《名醫別錄》		Y	N	Bitter in taste and cold in nature. Effects: clearing heat and toxicity, dispelling wind, and removing dampness. Used for treating encephalitis, enteritis, jaundice, sore throat, common cold and headache, measles, cough and asthma, mumps, tonsillitis, infectious hepatitis, dysentery, and stranguria.
16.	*Cyanthillium patulum* (Dryand. ex Dryand.) H.Rob.	Compositae	Whole plant	Liǔ zhīhuáng (柳枝癀)	1	1.82	—	B	Y	Y	Slightly bitter and acrid in taste and neutral in nature. Effects: clearing heat and promoting

	[*Vernonia patula* (Aiton) Merr.]										diuresis, removing blood stasis and swelling, detoxifying, and relieving diarrhea. Used for treating pathogenic wind and heat, common cold, headache, breast abscess, vomiting and diarrhea, dysentery, sore, furuncle, eczema, addiction rash, liver disease, acute gastroenteritis, and traumatic injury.
17.	*Dicliptera chinensis* (L.) Juss.	Acanthaceae	Whole plant	Gǒu gān cài (狗肝菜)	1	1.82	—	ABC	Y	Y	Slightly bitter in taste and cold in nature. Effects: clearing heat and toxicity, cooling blood, inducing diuresis, clearing liver heat, and promoting fluid production.

											Used for treating common cold, fever, furuncle, conjunctival congestion, swelling and pain, urination, and malaria.
18.	*Dimocarpus longan* Lour. [*Euphoria longana* Lam.]	Sapindaceae	Fruit	Lóng yǎn ròu (龍眼肉)	1	1.82	Shén nóng běn cǎo jīng《神農本草經》		Y	Y	Sweet in taste and warm in nature. Effects: benefiting heart and spleen, invigorating qi and blood, and tranquilizing mind. Used for treating asthenia, weakness, insomnia, amnesia, and severe palpitation.
19.	*Elephantopus scaber* L.	Compositae	Whole plant	Ding shù wū (丁豎杇)	2	3.64	Diān nán běn cǎo《滇南本草》	ABC	Y	Y	Bitter in taste and cool in nature. Effects: clearing heat and toxicity; promoting diuresis and detumescence. Used for treating common cold,

										dysentery, vomiting and diarrhea, tonsillitis, sore throat, edema, reddish eyes, furuncle, and swelling.
20.	*Eleutherococcus senticosus* (Rupr. & Maxim.) Maxim. [*Acanthopanax senticosus* (Rupr. et Maxim.) Harms]	Araliaceae	Stem	Cìwǔ jiā (刺五加)	1	1.82	—		N	N	Slightly bitter and acrid in taste and warm in nature. Effects: reinforcing kidney and strengthening waist; enriching qi and soothing; stimulating blood circulation and dredging collaterals. Used for weakness due to kidney deficiency, soreness and weakness of waist and knees, delayed walking in children, fatigue due to spleen deficiency, edema due to qi deficiency, loss

										of appetite, insomnia and dreaminess, forgetfulness, chest pain, arthralgia due to pathogenic wind, cold, and dampness, swelling and pain from falls.
21.	*Euphorbia thymifolia* L. [*Chamaesyce thymifolia* (L.) Millsp.]	Euphorbiaceae	Whole plant	Hóng rǔ zī cǎo (紅乳仔草)	1	1.82	Sheng cǎo yào xìng bèi yào《生草藥性備要》	Y	Y	Slightly sour and astringent in taste and slightly cool in nature. Effects: clearing heat and toxicity, promoting diuresis, and relieving itching. Used for treating bacillary dysentery, enteritis diarrhea, and hemorrhage caused by hemorrhoids.
22.	*Ficus formosana* Maxim.	Moraceae	Root	Xiǎo hào niú nǎi pǔ	1	1.82	—	Y	Y	Sweet and slightly astringent in taste and bland in nature.

				(小號牛奶埔)						Effects: softening liver and spleen, clearing heat, and promoting diuresis. Used for treating acute and chronic hepatitis, lumbar muscle sprain, edema, and urinary stranguria.
23.	*Glossocardia bidens* (Retz.) Veldkamp	Compositae	Whole plant	Fēng rú cǎo (風茹草)	9	16.36	—	Y	Y (Penghu County)	Slightly acrid, cool, sweet, and slightly bitter in taste. Effects: clearing heat and toxicity, promoting diuresis and detumescence, promoting blood circulation and removing blood stasis. Used for treating heatstroke, vomiting and diarrhea, common cold and fever, damp-heat edema, dysentery,

											gingivitis, backache, acute tonsillitis, bronchitis, enteritis, diarrhea, and urethritis. Topical application is performed by applying the fresh product to the affected part. It is used to treat traumatic injury, snakebite, herpes zoster, carbuncle, sore, furuncle, and traumatic injury.
24.	*Glycyrrhiza uralensis* Fisch.	Leguminosae	Root & rhizome	Gān cǎo (甘草)	8	14.55	Shén nóng běn cǎo jīng《神農本草經》	A	N	N	Sweet in taste and neutral in nature. Effects: tonifying qi and strengthening the middle, relieving spasm and pain, moistening lung, and relieving cough. Used in clearing heat and detoxifying and harmonizing

										various drugs. Applied for treating burnout and reduced food intake, thin skin and yellow face, palpitation and shortness of breath, abdominal pain, loose stool, limbs spasm, acute pain, hysteria, cough and asthma, sore throat, carbuncle, sore, swelling and pain, fetus toxin, and drug and food poisoning
25.	*Hordeum vulgare* L.	Poaceae	Seed	Mài yá (麥芽)	1	1.82	Yào xìng lùn《藥性論》		Y	Y	Sweet in taste and neutral in nature. Effects: invigorating spleen and stimulating appetite, promoting qi circulation and digestion, suppressing lactation and relieving

											distension. Used for treating dyspepsia, abdominal distention and pain, spleen deficiency, anorexia, breast stasis, breast distention and pain, and weaning of women.
26.	*Houttuynia cordata* Thunb.	Saururaceae	Whole plant	Yú xīng cǎo (魚腥草)	14	25.45	Míng yī bié lù《名醫別錄》	ABC	Y	Y	Acrid and sour in taste and cool in nature. Effects: clearing heat and toxicity, eliminating carbuncle, expelling pus, and inducing diuresis. Used for treating lung abscess, cough from lung heat, stranguria, and edema.
27.	*Ilex asprella* (Hook. & Arn.) Champ. ex Benth.	Aquifoliaceae	Stem & Root	Wàn diǎn jīn (萬點金)	14	25.45	Shengcǎoyàoxìngbèiyào《生草藥性備要》	AC	Y	N	Bitter and sweet in taste and cold in nature. Effects: clearing heat and toxicity,

											promoting fluid production to quench thirst, and promoting blood circulation. Used for treating common cold, lung abscess, breast moth, sore throat, stranguria, toothache from wind and fire, scrofula, carbuncle, furuncle, allergic dermatitis, bleeding due to hemorrhoids, snakebite, and traumatic injury.
28.	*Imperata cylindrica* (L.) Raeusch.* [*Imperata cylindrica* (L.) P. Beauv. var. *major* (Nees) C. E. Hubb.]	Poaceae	Rhizome	Bái máo gēn (白茅根)	4	7.27	Shén nóngběn cǎo jīng《神農本草經》	ABC	Y	N	Sweet in taste and cold in nature. Effects: cooling blood to stop bleeding, clearing heat, and inducing diuresis. Used for treating measles, fever, polydipsia,

										hematemesis, and edema.
29.	*Ixeris chinensis* (Thunb. ex Thunb.) Nakai	Compositae	Whole plant	Xiǎo jīn yīng (小金英)	2	3.64	Zhí wùmíng shítúkǎo《植物名實圖考》	Y	Y	Bitter in taste and cool in nature. Effects: clearing heat and toxicity, reducing heat, cooling blood, stopping bleeding, relieving pain, regulating menstruation, promoting blood circulation, removing necrotic tissue, and promoting granulation. Used for treating innominate toxic swelling, scrotal eczema, wind-heat cough, diarrhea, dysentery, hematemesis, epistaxis, impetigo, traumatic injury, fracture, pneumonia, pulmonary

											abscess, urethral calculus, and snakebite.
30.	*Justicia procumbens* L.	Acanthaceae	Whole plant	Shǔ wěi huáng (鼠尾癀)	2	3.64	Shén nóng běn cǎo jīng《神農本草經》	B	Y	Y	Salty and acrid in taste and cold in nature, with minor toxicity. Effects: clearing heat and toxicity, promoting diuresis, eliminating stagnation, promoting blood circulation, and relieving pain. Used for treating common cold, fever, dysentery, jaundice, and traumatic injury.
31.	*Kadsura japonica* (L.) Dunal	Schisandraceae [Magnoliaceae]	Stem	Hóng gǔ shé (紅骨蛇)	7	12.73	—		Y	N	Acrid, astringent, and bitter in taste and neutral in nature. Effects: relieving fever, quenching thirst, relieving pain, dispelling wind, relaxing muscles and tendons, cooling blood,

										stopping dysentery, reducing swelling, and promoting blood circulation. Used for treating rheumatism and traumatic injury.
32.	*Lophatherum gracile* Brongn.	Poaceae	Whole plant	Dànzhúyè (淡竹葉)	2	3.64	Diānnánběn cǎo《滇南本草》	ABC	Y	N	Sweet in taste and light and cold in nature. Effects: clearing heat, promoting diuresis, and relieving restlessness. Used for treating fever, polydipsia, difficult urination, dark urine, stranguria, and aphtha in the mouth or on the tongue.
33.	*Lycium chinense* Mill.	Solanaceae	Fruit	Gǒu qǐ zǐ (枸杞子)	2	3.64	Shén nóng běn cǎo jīng《神農本草經》	C	Y	Y	Sweet in taste and neutral in nature. Effects: nourishing kidney, moistening lung, nourishing liver,

								and improving eyesight. Used for treating yin deficiency of liver and kidney, soreness and weakness of waist and knees, dizziness, diabetes, and spermatorrhea.
34.	Root	Gǒu qǐ gēn (枸杞根)	1	1.82	Shén nóng běn cǎo jīng《神農本草經》	Y	Y	Sweet in taste and cold in nature. Effects: clearing deficiency heat, clearing lung heat, and cooling blood. Used for treating yin deficiency and fatigue, hectic fever and night sweat, infantile malnutrition and fever, lung heat, asthma and cough, hematemesis, epistaxis, hematuria, and diabetes.

35.	*Mallotus repandus* (Willd.) Muell.-Arg.	Euphorbiaceae	Stem	Tǒng jiāo téng (桶交藤)	4	7.27	Zhí wùmíngshítúkǎo《植物名實圖考》		Y	N	Sweet and slightly bitter in taste and cold in nature. Effects: dispelling wind and dampness; promoting blood circulation and dredging collaterals; detoxifying and detumescence; and expelling parasites and relieving itching. Used for treating rheumatic arthralgia, chronic ulcer, snakebite, ascariasis, traumatic injury, bloated sore, eczema, rheumatic arthritis, lumbago and leg pain, and puerperal paralysis.
36.	*Mentha arvensis* L.	Lamiaceae	Whole plant	Bó hé (薄荷)	22	40.00	Léigongpáozhìlùn《雷公炮炙論》	ABC	Y	Y	Acrid in taste and cool in nature, with fragrance.

										Effects: clearing heat and relieving exterior syndrome, relieving sore throat and rash, invigorating stomach, relieving swelling and itching. Used for treating wind-heat common cold, headache, pharyngolaryngitis, cough, early stage of measles, dyspepsia, flatulence in the heart, and skin pruritus.
37. *Morus alba* L.	Moraceae	Leaf	Sāng yè (桑葉)	5	9.09	Shén nóngběn cǎo jīng《神農本草經》	ABC	N	Y	Bitter and sweet in taste and cold in nature. Effects: dispelling wind, clearing heat, clearing liver, and improving eyesight. Used for treating wind-heat common cold,

										cough due to lung heat dryness, headache, dizziness, dim eyesight, edema, and sore throat.
38.			Twig	Sāng zhī (桑枝)	3	5.45	Běncǎotújīng《本草圖經》	N	Y	Bitter in taste and neutral in nature. Effects: dispelling wind and clearing heat; dredging collaterals and benefiting joints. Used for treating shoulder and arm joint pain, and numbness.
39.	*Mosla chinensis* Maxim.	Lamiaceae	Aerial parts	Xiāng rú (香薷)	2	3.64	Míng yībiélù《名醫別錄》	Y	N	Acrid in taste and mildly warm in nature. Effects: promoting sweating to relieve summer heat, harmonizing the middle, and removing dampness stagnation and swelling. Used

										for treating exogenous wind-cold in summer, internal injuries due to dampness, cold aversion and fever, headache and no sweat, abdominal pain, vomiting, diarrhea, dysuria, and edema.
40.	*Mucuna macrocarpa* Wall.	Leguminosae	Stem	Xiě téng (血藤)	4	7.27	—	Y	N	Bitter and astringent in taste and mildly warm in nature. Effects: relaxing tendons and activating collaterals, stimulating blood circulation, clearing lung-heat and moistening dryness, and regulating menstruation. Used for treating rheumatic arthralgia, polio

										sequelae, irregular menstruation, anemia, lung heat, dry cough, hemoptysis, soreness of waist and knees, rheumatic arthralgia, numbness of hands and feet, and paralysis.
41.	*Ocimum basilicum* L.	Lamiaceae	Whole plant	Jiǔ céng tǎ (九層塔)	1	1.82	Shān hǎi jīng《山海經》	Y	Y	Acrid and sweet in taste and warm in nature. Effects: wind dispelling and relieving exterior syndrome, dehumidifying and neutralizing; promoting qi, blood circulation, detoxification, and detumescence. Used for treating cold headache, fever and cough, heat stroke, dyspepsia, loss

											of appetite, abdominal distension and pain, vomiting, diarrhea, rheumatic arthralgia, spermatorrhea, irregular menstruation, toothache and halitosis, pterygium (covering the eye), wet skin sores, addiction rash and pruritus, traumatic injuries, and snakebite.
42.	*Ocimum gratissimum* L.	Lamiaceae	Stem	Shān jiǔ céng tǎ (山九層塔)	8	14.55	—	B	Y	Y	Acrid in taste and warm in nature. Used for treating rheumatism and backache.
43.	*Oldenlandia diffusa* (Willd.) Roxb.*	Rubiaceae	Whole plant	Bái huā shé shé cǎo (白花蛇舌草)	3	5.45	—	ABC	Y	Y	Bitter and sweet in taste and cold in nature. Effects: clearing heat and toxicity, promoting

	[*Hedyotis diffusa* Willd.]										diuresis, eliminating carbuncle, and resisting cancer. Used for treating malignant tumor, intestinal carbuncle, sore throat, damp-heat jaundice, dysuria, sore, furuncle, toxic swelling, and venomous snakebite.
44.	*Onychium japonicum* (Thunb.) Kunze	Pteridaceae	Whole plant	Fèng wěi lián (鳳尾連)	1	1.82	Zhíwùmíngshítúkǎo 《植物名實圖考》		Y	Y	Bitter in taste and cold in nature. Effects: clearing heat, promoting diuresis, detoxifying, and stopping bleeding. It is used as a bitter stomachic for dysentery and gastroenteritis.
45.	*Orthosiphon aristatus* (Blume) Miq.	Lamiaceae	Whole plant	Huà shícǎo (化石草)	2	3.64	—	BC	Y	Y	Sweet and bland in tasteslightly bitter and cool in nature. Effects: clearing heat,

											inducing diuresis, and removing calculi. Used for treating acute and chronic nephritis, cystitis, urinary calculi, and rheumatic arthritis.
46.	*Perilla frutescens* (L.) Britton	Lamiaceae	Leaf	Zǐ sū (紫蘇)	2	3.64	Míng yībiélù《名醫別錄》	AB	Y	Y	Acrid in taste and warm in nature. Effects: relieving exterior syndrome, dispelling cold, resolving food stagnation, promoting and regulating qi circulation, and harmonizing stomach. Used for treating common cold, cough, vomiting, asthma, dyspepsia, and fish and crab poisoning.

47.	*Plantago asiatica* L.	Plantaginaceae	Whole plant	Chē qián cǎo (車前草)	5	9.09	Shī jīng《詩經》	ABC	Y	Y	Sweet in taste and cold in nature. Effects: clearing heat and diuresis, eliminating phlegm, cooling blood, and detoxifying. Used for treating edema, scanty urine, heat stranguria, astringent pain, dysentery due to summer heat and dampness, cough due to phlegm heat, hematemesis, epistaxis, carbuncle, swelling, and sore.
48.	*Platostoma palustre* (Blume) A.J.Paton [*Mesona chinensis* Benth.]	Lamiaceae	Whole plant	Xiān cǎo (仙草)	40	72.73	Běn cǎo gāng mùshíyí《本草綱目拾遺》	AC	Y	Y	Sweet in taste and cool in nature. Effects: clearing heat, quenching thirst, cooling blood, relieving heat, and lowering blood pressure. Used for treating

											heatstroke, common cold, muscle pain, arthralgia, hypertension, gonorrhea, kidney disease, visceral fever, and diabetes.
49.	*Pogonatherum crinitum* (Thunb.) Kunth	Poaceae	Whole plant	Bǐ zī cǎo (筆仔草)	8	14.55	Běn cǎo gāng mù《本草綱目》	ABC	Y	Y	Sweet and bland in tasteand cold in nature. Effects: clearing heat and toxicity, inducing diuresis, cooling blood, and resisting cancer. Used for treating icteric hepatitis, fever, polydipsia, stranguria by inducing diuresis, dysuria, hematuria, and diabetes.
50.	*Prunella vulgaris* L.	Lamiaceae	Spike	Xià kū cǎo (夏枯草)	4	7.27	Shén nóngběn cǎo jīng《神農本草經》	ABC	Y	Y	Bitter and acrid in taste and cold in nature. Effects: clearing liver, resolving hard masses, and

											reducing swelling. Used for treating conjunctival congestion, swelling and pain, conjunctival night pain, headache, vertigo, scrofula, goiter, breast abscess, hyperplasia of mammary glands, and hypertension.
51.	*Pteris multifida* Poir.	Pteridaceae	Whole plant	Fèng wěi cǎo (鳳尾草)	24	43.6 4	Běn cǎoshíyí《本草拾遺》	C	Y	Y	Bitter in taste and slightly cold in nature. Effects: clearing heat and promoting diuresis, cooling blood and detoxifying. Used for treating dysentery, hepatitis, urethritis, hemoptysis, toothache, and stomatitis.

52.	*Rhinacanthus nasutus* (L.) Kurz	Acanthaceae	Whole plant	Bái hè líng zhī (白鶴靈芝)	16	29.09	—	B	N	Y	Sweet, bland, and slightly bitter in taste, mild in nature, and slightly toxic. Effects: moistening lungs for relieving cough, calming liver, reducing fire, promoting detumescence, detoxification, disinfestation and antipruritic effects. Used for treating hypertension, diabetes, liver disease, pulmonary tuberculosis, damp-heat in spleen and stomach, and eczema.
53.	*Salvia plebeia* R. Br.	Lamiaceae	Whole plant	Qī céng tǎ (七層塔)	6	10.91	Běn cǎogāng mù《本草綱目》		Y	Y	Bitter and acrid in taste and cool in nature. Effects: clearing heat;promoting detoxification and diuresis. Used for treating

											sore throat, carbuncle, swollen sore, breast carbuncle, sore pain, hemorrhage, cough, phlegm and asthma, hemoptysis, hematemesis, edema, abdominal distension, traumatic injury, and snake and dog bites.
54.	*Sambucus javanica* Blume [*Sambucus chinensis* Lindl.]	Adoxaceae [Caprifoliaceae]	Stem	Mǒu gǔ xiāo (冇骨消)	1	1.82	Shén nóngběn cǎo jīng《神農本草經》		Y	N	Sweet and sour in taste, mild in nature, and slightly toxic. Effects: promoting detumescence, detoxification, diuresis, and blood circulation; and removing blood stasis. Antipyretic and analgesic. Used for treating pulmonary abscess,

											rheumatic arthritis, nameless swelling, beriberi edema, diarrhea, jaundice, cough, and phlegm asthma. Topical use for traumatic injury and fracture.
55.	*Scaphium affine* (Mast.) Pierre [*Sterculia lychnophora* Hance]	Malvaceae [Sterculiaceae]	Seed	Péng dà hǎi (膨大海)	1	1.82	Běn cǎogāng mùshí yí《本草綱目拾遺》	A	N	N	Sweet and bland in tasteand cool in nature. Effects: clearing heat and moistening lungs, relieving pharynx, clearing bowels, and relieving constipation. Used for treating dry cough without phlegm, sore throat, dull voice, toothache, and constipation due to heat accumulation.

56.	*Scoparia dulcis* L.	Plantaginaceae [Scrophulariaceae]	Whole plant	Zhūzīcǎo (珠仔草)	5	9.09	—	B	Y	Y	Sweet in taste and neutral in nature. Effects: clearing heat and toxicity, inducing diuresis to reduce swelling, promoting fluid production to quench thirst, dispelling wind, and relieving itching. Used for treating cough due to lung heat, exogenous wind-heat, diarrhea, dysentery, dysuria, infantile malnutrition, beriberi, eczema, infantile measles, hot miliaria, sore throat, erysipelas, snake injury, and preventing heatstroke and reddish eyes.

57.	*Scutellaria barbata* D. Don	Lamiaceae	Whole plant	Bàn zhī lián (半枝蓮)	2	3.64	—	BC	Y	Y	Acrid in taste and neutral in nature. Effects: clearing heat and toxicity, promoting blood circulation and removing blood stasis, relieving swelling and pain, and resisting cancer. Used for treating hematemesis, jaundice, cancer, traumatic injury, and venomous snakebite.
58.	*Sida rhombifolia* L.	Malvaceae	Whole plant	Sìmǐcǎo (賜米草)	1	1.82	—		Y	N	Sweet and acrid in taste and cool in nature. Effects: clearing heat, promoting diuresis and blood circulation, and expelling pus. Used for treating influenza, tonsillitis, dysentery, diarrhea, jaundice, hemorrhoids,

										hematemesis, carbuncle, and bile furuncle.
59.	*Sigesbeckia orientalis* L.	Compositae	Aerial parts	Kǔ cǎo (苦草) Xī liàn cǎo (豨薟草)	1	1.82	Xīnxiūběn cǎo《新修本草》	Y	N	Bitter in taste and cold in nature. Effects: dispelling wind and dampness, benefiting bones and muscles, and lowering blood pressure. Used for treating rheumatic arthritis, numbness of limbs, weakness of waist and knees, hemiplegia, and hepatitis.
60.	*Solanum americanum* Mill. [*Solanum nigrum* L.]	Solanaceae	Whole plant	Lóng kuí (龍葵)	1	1.82	Yào xìng lùn《藥性論》	Y	N	Bitter in taste, slightly sweet, and cold in nature, with small toxicity. Effects: clearing heat and toxicity, reducing swelling and resolving hard mass, promoting blood

										circulation, and inducing diuresis. Used for treating carbuncle, erysipelas, cancer, furuncle, traumatic injury, chronic cough and asthma, and edema.
61.	*Solanum capsicoides* All.	Solanaceae	Stem	Cìqié (刺茄)	1	1.82	—	Y	N	Bitter and acrid in taste, warm and toxic. Effects: relieving cough and asthma, dispelling blood stasis, and relieving pain. Used for treating asthma, rheumatism, stomachache, traumatic injury, and snakebite.
62.	*Solanum incanum* L.	Solanaceae	Stem	Huáng shuǐ qié (黃水茄)	1	1.82	—	Y	Y	Bitter in taste and cold in nature, toxic. Effects: anti-inflammatory effect, detoxifying,

										expelling wind, relieving pain, clearing heat, and diminishing inflammation. Used for treating headache, toothache, pharyngalgia, stomachache, rheumatic arthralgia, traumatic injury, carbuncle, sore, swelling, hepatitis, liver cirrhosis, lymphadenitis, pleurisy, edema, sinusitis, and eye diseases.
63.	*Solanum violaceum* Ortega	Solanaceae	Stem	Liǔzīqié (柳仔茄)	2	3.64	—	Y	Y	Bitter in taste and neutral in nature, with mild toxicity. Effects: clearing heat and dampness, removing blood stasis, and reducing swelling. Used for treating rheumatic arthralgia,

											furuncle, abdominal pain, headache, toothache, sore throat, tonsillitis, malnutrition, traumatic injury, and scrofula.
64.	*Sphagneticola calendulacea* (L.) Pruski [*Wedelia chinensis* (Osbeck) Merr.]	Compositae	Whole plant	Huáng huā mì cài (黃花蜜菜)	20	36.36	Shengcǎo yàoxìngbèiyào《生草藥性備要》	B	Y	Y	Sweet and bland in tasteand cool in nature. Effects: clearing heat and toxicity, removing blood stasis, and reducing swelling. Used for treating diphtheria, cough, whooping cough, dysentery, hemorrhoids, and traumatic injury.
65.	*Stevia rebaudiana* (Bertoni) Bertoni	Compositae	Leaf	Tiánjú (甜菊)	1	1.82	—		N	Y	Sweet in taste and neutral in nature. Effects: strengthening body, harmonizing stomach,

											contraception, promoting fluid production, relieving cough, and lowering blood pressure. Used for treating diabetes and hypertension.
66.	*Taraxacum campylodes* G.E.Haglund * [*Taraxacum officinale* Weber ex Wiggers]	Compositae	Whole plant	Pú gōng yīng (蒲公英)	1	1.82	Xīnxiūběn cǎo《新修本草》	AC	Y	Y	Bitter and sweet in taste and cold in nature. Effects: clearing heat and toxicity, inducing diuresis, eliminating carbuncle, and resolving hard mass. Used for treating upper respiratory tract infection, acute tonsillitis, conjunctivitis, epidemic parotitis, acute mastitis, gastritis, enteritis, dysentery, hepatitis, cholecystitis, acute

											appendicitis, urinary system infection, disc cavity inflammation, carbuncle, furuncle, and snake and insect bite.
67.	*Tithonia diversifolia (Hemsl.)* A. Gray	Compositae	Stem	Wǔzhǎojīnyīng (五爪金英)	3	5.45	—		Y	Y	None
68.	*Tradescantia spathacea* Sw. [*Rhoeo discolor* Hance]	Commelinaceae	Leaf	Banglán (蚌蘭)	1	1.82	—	AB	N	Y	Sweet and bland in tasteand cool in nature. Effects: clearing heat, moistening lung, eliminating phlegm, relieving cough, cooling blood, stopping bleeding, removing blood stasis, stopping dysentery, and relieving depression. Used for treating pneumonia, dry cough due to lung heat,

											hematemesis due to overexertion injury, and traumatic injury.
69.	*Tridax procumbens* (L.) L.	Compositae	Whole plant	Fèi yáncǎo (肺炎草)	1	1.82	—		Y	N	Bitter in taste and cool in nature. Antipyretic and anti-inflammatory effects. Used for treating pneumonia, cough, cold, and high fever.
70.	*Vitis thunbergii* Sieb. & Zucc.	Vitaceae	Stem	Xiǎoběnshān pú tao (小本山葡萄)	1	1.82	Běn cǎo gāng mù《本草綱目》		Y	Y	Sweet in taste and neutral in nature. Effects: detoxifying, promoting diuresis, and eliminating dampness. Used for treating rheumatism, jaundice, and gonorrhea.
71.	*Ziziphus jujuba* Mill.	Rhamnaceae	Fruit	Dàzǎo (大棗)	2	3.64	Shén nóngběn cǎo jīng《神農本草經》	A	N	Y (Miaoli County)	Sweet in taste and warm in nature. Effects: calming heart, nourishing liver,

	soothing the mind, and sweating. Used for treating insomnia, palpitations due to fright, polydipsia, and deficiency in sweating.

Regional comparison codes: A, Lingnan (Liu et al., 2013b); B, Chaoshan (Li et al., 2017); C, Fujian (Lin, 2014).Each scientific namemarked with an asterisk (*) indicates the use of multiple sources of medicinal materials: *Artemisia capillaris* Thunb. and *Origanum vulgare* L.; *Astragalus propinquus* Schischkin and *Hedysarum polybotrys* Hand.-Mazz.; *Imperata cylindrica* (L.) Raeusch. and *Pennisetum flaccidum* Griseb.; *Oldenlandia diffusa* (Willd.) Roxb. and *Oldenlandia corymbosa* L.; *Taraxacum campylodes* G.E.Haglund and *Ixeris chinensis* (Thunb.) Nakai.

The scientific names (1) and plant family names (2) in Table A.1 are based on "The Plant List (www.theplantlist.org)". The scientific name in "[*genus species*]"means that the scientific name often appears in pharmacopoeia and botanical records (the "Taiwan Herbal Pharmacopoeia," 3rd Edition, the Enumeratio Plantarum Formosanarum, 2nd Edition, and the Taiwan Biodiversity Information Facility (http://taibif.tw/))

Appendix B

Table B.1

General properties and pharmacological effects of highly cited raw materials of qīng-cǎo-chá tea (UV>5).

No.	Scientific name	Family	Parts used	Local name	UV (%)	Literature on medicinal properties and effects (PubMed)
1.	*Platostoma palustre* (Blume) A.J.Paton	Lamiaceae	Whole plant	Xiān cǎo (仙草)	72.73	Antidiabetic (Adisakwattana et al., 2014; Chusak et al., 2014; Liu et al., 2018; Yuris et al., 2018) and antioxidative (Lin et al., 2017; Tang et al., 2017) effects.
2.	*Bidens pilosa* L.	Compositae	Whole plant	Xián fēng cǎo (咸豐草)	49.09	Anticancer (Wu et al., 2013), gastroprotective (Alvarez et al., 1999; Horiuchi et al., 2010), antidiabetic (Chien et al., 2009; Ubillas et al., 2000), anti-allergic (Matsumoto et al., 2009), and anti-inflammatory (Yoshida et al., 2006) effects.
3.	*Pteris multifida* Poir.	Pteridaceae	Whole plant	Fèng wěi cǎo (鳳尾草)	43.64	Anticancer (Kim et al., 2017b), anti-neuroinflammatory (Kim et al., 2017c), anti-hyperlipidemic (Wang et al., 2010), and antioxidative (Wang et al., 2007) effects.
4.	*Mentha arvensis* L.	Lamiaceae	Whole plant	Bóhé (薄荷)	40.00	Antimicrobial (de Sousa Guedes et al., 2018; Viji et al., 2015), antidiabetic (Agawane et al., 2017), anti-stress (Tian et al., 2018), anti-asthmatic (Sharma et al., 2018), anticancer (Sharma et al., 2014), and antioxidative (Ahmad et al., 2012) effects.

5.	*Sphagneticola calendulacea* (L.) Pruski	Compositae	Whole plant	Huáng huā mì cài (黃花蜜菜)	36.36	Antidiabetic (Thao et al., 2018), anticancer (Huang et al., 2016; Lin et al., 2007; Liu et al., 2013a; Tsai et al., 2009, 2015, 2017a, 2017b), gastroprotective (Huang et al., 2013; Wei et al., 2017), neuroprotective (Lin et al., 2007), antibacterial (Darah et al., 2013), anti-inflammatory (Darah et al., 2013), and antioxidative (Manjamalai and Berlin Grace, 2012) effects.
6.	*Rhinacanthus nasutus* (L.) Kurz	Acanthaceae	Whole plant	Bái hèlíng zhī (白鶴靈芝)	29.09	Anticancer (Boueroy et al., 2018; Horii et al., 2012; Kupradinun et al., 2009; Siripong et al., 2006, 2009, 2012), anti-obesity (Ngoc et al., 2019), antiglycemic (Shah et al., 2017), neuroprotective (Brimson et al., 2011, 2012; Chang et al., 2016; Chuang et al., 2017), acethylcholinesterase-inhibitory (Boonyaketgoson et al., 2017), antioxidative (Brimson et al., 2012; Shah et al., 2017), antidiabetic (Shah et al., 2017; Visweswara Rao et al., 2013a), neuraminidase-inhibitory (Kwak et al., 2018), hepatoprotective (Visweswara Rao et al., 2013b), anti-inflammatory (Tewtrakul et al., 2009b), antimicrobial (Kernan et al., 1997; Ngoc et al., 2019; Puttarak et al., 2010), anti-allergic (Tewtrakul et al., 2009a), and immunomodulatory (Punturee et al., 2005) effects.
7.	*Artemisia capillaris* Thunb.	Compositae	Whole plant	Yīn chén (茵陳)	25.45	Gastroprotective (Vouillamoz et al., 2015), alcohol metabolism-stimulating (Vouillamoz et al., 2015), antidiabetic (Vouillamoz et al., 2015), hepatoprotective (Jang et al., 2015), anti-infective (Abad et al., 2012) effects.

8.	*Houttuynia cordata* Thunb.	Saururaceae	Whole plant	Yú xīng cǎo (魚腥草)	25.45	Anti-anaphylactic, antimutagenic, anti-inflammatory, antiviral, anti-obesity, antibacterial, anticancer, anti-allergic, antidiabetic, and antioxidative effects (Kumar et al., 2014).
9.	*Ilex asprella* (Hook. & Arn.) Champ. & Benth.	Aquifoliaceae	Stem & root	Wàndiǎnjīn (萬點金)	25.45	Anticancer (Li et al., 2018), immunoregulatory (Meng et al., 2018), anti-inflammatory (Yang et al., 2018), anti-hyperlipidemic (Hu et al., 2012), and antiviral (Zhou et al., 2012) effects, along with acute respiratory distress syndrome relief (Dai et al., 2014).
10.	*Glossocardia bidens* (Retz.) Veldkamp	Compositae	Whole plant	Fēng rú cǎo (風茹草)	16.36	Anti-inflammatory (Houng et al., 2017), anti-hyperlipidemic (Lee et al., 2016), anti-atherosclerotic (Hsuan et al., 2015), hepatoprotective (Tien et al., 2014), antioxidative (Yang et al., 2006), antimicrobial (Yang et al., 2014), anticancer (Hsu et al., 2008), immunomodulatory (Ha et al., 2006), and anti-inflammatory (Wu et al., 2005) effects.
11.	*Glycyrrhiza uralensis* Fisch.	Leguminosae	Root & rhizome	Gān cǎo (甘草)	14.55	Antitussive effects as an expectorant, along with antiulcer, antispasmodic, hepatoprotective, anti-inflammatory, anti-allergic, and antimicrobial effects (World Health Organization, 1999).
12.	*Ocimum gratissimum* L.	Lamiaceae	Stem	Shānjiǔ céng tǎ (山九層塔)	14.55	Antibacterial (Chimnoi et al., 2018; Talabi and Makanjuola, 2017), antifungal (Mohr et al., 2017), renal-protective (Ogundipe et al., 2017), antidiabetic (Akpan et al., 2014; Okoduwa et al., 2017), anti-obesity (Chao et al., 2017; Irondi et al., 2016), antioxidative (Ajayi et al., 2017b; Okon and Umoren, 2017), anti-inflammatory (Ajayi et al., 2017a, 2017b), anti-hyperlipidemic (Chao et al., 2016), hepatoprotective (Chen et al., 2015),

						antitrypanosomal (Kpadonou Kpoviessi et al., 2014), anticancer (Ekunwe et al., 2013; Lin et al., 2014), antiulcer (Ofem et al., 2012), antiplaque (Pereira et al., 2011), antigingivitis (Pereira et al., 2011), neuroprotective (Bora, 2011), anticonvulsant (Okoli et al., 2010), and antianxiety (Okoli et al., 2010) effects, along with beneficial effects in the management of erectile dysfunction (Ojo et al., 2019) and neuropathic pain (Paula-Freire et al., 2016).
13.	*Pogonatherum crinitum* (Thunb.) Kunth	Poaceae	Whole plant	Bǐzīcǎo (筆仔草)	14.55	Anti-inflammatory effects (Wang et al., 2008).
14.	*Kadsura japonica* (L.) Dunal	Schisandraceae	Stem	Hónggǔshé (紅骨蛇)	12.73	Antihepatitis activity (Kuo et al., 2005).
15.	*Salvia plebeia* R. Br.	Lamiaceae	Whole plant	Qīcéngtǎ (七層塔)	10.91	Anti-inflammatory (Bonesi et al., 2017; Jang et al., 2016), antioxidative (Bonesi et al., 2017), anti-hyperuricemia (Kim et al., 2017a), anti-influenza (Bang et al., 2018), anti-obesity (Choi et al., 2016), and antiviral (Bang et al., 2016) effects, along with beneficial effects in suppressing osteoclastogenesis (Kim et al., 2016).
16.	*Morus alba* L.	Moraceae	Leaf	Sāngyè (桑葉)	9.09	Antidiabetic, immunomodulatory, anti-inflammatory, antioxidative, hepatoprotective, renoprotective, and anti-obesity effects, along with beneficial effects in regulating the gut microbiota and decreasing both rate and extent of lipid digestion (He et al., 2018).

17.	*Plantago asiatica* L.	Plantaginaceae	Whole plant	Chē qiáncǎo (車前草)	9.09	Immunomodulatory (Chiang et al., 2003; Yin et al., 2019), anti-hyperlipidemic (Yang et al., 2017), renoprotective (Kho et al., 2017), antiglycemic (Choi et al., 2008), antioxidative (Choi et al., 2008), and antiviral (Chiang et al., 2003) effects.
18.	*Scoparia dulcis* L.	Plantaginaceae	Whole plant	Zhūzīcǎo (珠仔草)	9.09	Antidiabetic, antioxidative, anti-inflammatory, analgesic, antimalarial, hepatoprotective, sedative-hypnotic, antiulcer, antisickling, and antimicrobial effects (Pamunuwa et al., 2016).
19.	*Imperata cylindrica* (L.) Raeusch.	Poaceae	Rhizome	Bái máogēn (白茅根)	7.27	None
20.	*Mallotus repandus* (Willd.) Muell.-Arg.	Euphorbiaceae	Stem	Tǒngjiāoténg (桶交藤)	7.27	Analgesic (Hasan et al., 2014), anti-inflammatory (Hasan et al., 2014), and antioxidative (Lin et al., 1995) effects.
21.	*Mucuna macrocarpa* Wall.	Leguminosae	Stem	Xiěténg (血藤)	7.27	Antileukemic effects (Lu et al., 2010).
22.	*Prunella vulgaris* L.	Lamiaceae	Spike	Xià kū cǎo (夏枯草)	7.27	Antimicrobial, anti-inflammatory, immunomodulatory, anticancer, antioxidative, antidiabetic effects, along with regulatory effects on blood pressure (Bai et al., 2016).
23.	*Clerodendrum cyrtophyllum* Turcz.	Lamiaceae	Root &stem	Guān yīn chuàn (觀音串)	5.45	Antioxidative effects (Zhou et al., 2013).

24.	*Oldenlandia diffusa* (Willd.) Roxb.	Rubiaceae	Whole plant	Bái huā shé shé cǎo (白花蛇舌草)	5.45	Anticancer, immunomodulatory, antioxidative, and anti-inflammatory effects (Chen et al., 2016).
25.	*Morus alba* L.	Moraceae	Twig	Sāngzhī (桑枝)	5.45	Antidiabetic, immunomodulatory, anti-inflammatory, antioxidative, hepatoprotective, renoprotective, and anti-obesity effects, along with beneficial effects in regulating the gut microbiota and decreasing both rate and extent of lipid digestion (He et al., 2018).
26.	*Tithonia diversifolia* (Hemsl.) A. Gray	Compositae	Stem	Wǔzhǎojī nyīng (五爪金英)	5.45	Immunomodulatory, analgesic, antimalarial effect, antidiabetic, antimicrobial, antioxidative, anticancer, anti-obesity, anti-hyperlipidemic, gastroprotective, antiemetic, antidiarrheal, antileishmanial, antitrypanosomal, and hepatoprotective effects, along with repellent and antivenin-like activities (Mabou Tagne et al., 2018).
